# Differences Between Central Venous and Cerebral Tissue Oxygen Saturation in Anaesthetised Patients With Diabetes Mellitus

**DOI:** 10.1038/s41598-019-56221-4

**Published:** 2019-12-24

**Authors:** Roberta Sudy, Ferenc Petak, Almos Schranc, Szilvia Agocs, Ivett Blaskovics, Csaba Lengyel, Barna Babik

**Affiliations:** 1 Department of Anaesthesiology and Intensive Therapy, University of Szeged, Szeged, Hungary; 2 Department of Medical Physics and Informatics, University of Szeged, Szeged, Hungary; 3 1st Department of Internal Medicine, Faculty of Medicine, University of Szeged, Szeged, Hungary

**Keywords:** Health care, Medical research

## Abstract

The brain has high oxygen extraction, thus the regional cerebral tissue oxygen saturation (rSO_2_) is lower than the central venous oxygen saturation (ScvO_2_). We hypothesised that diabetes widens the physiological saturation gap between ScvO_2_ and rSO_2_ (gSO_2_), and the width of this gap may vary during various phases of cardiac surgery. Cardiac surgery patients with (n = 48) and without (n = 91) type 2 diabetes mellitus (T2DM) underwent either off-pump coronary artery bypass (OPCAB) or other cardiac surgery necessitating cardiopulmonary bypass (CPB) were enrolled. rSO_2_ was measured by near-infrared spectroscopy (NIRS) and ScvO_2_ was determined simultaneously from central venous blood. rSO_2_ was registered before and after anaesthesia induction and at different stages of the surgery. ScvO_2_ did not differ between the T2DM and control patients at any stage of surgery, whereas rSO_2_ was lower in T2DM patients, compared to the control group before anaesthesia induction (60.4 ± 8.1%[SD] vs. 67.2 ± 7.9%, p<0.05), and this difference was maintained throughout the surgery. After anaesthesia induction, the gSO_2_ was higher in diabetic patients undergoing CPB (20.2 ± 10.4% vs. 12.4 ± 8.6%, p < 0.05) and OPCAB grafting surgeries (17.0 ± 7.5% vs. 9.5 ± 7.8%, p < 0.05). While gSO_2_ increased at the beginning of CPB in T2DM and control patients, no significant intraoperative changes were observed during the OPCAB surgery. The wide gap between ScvO_2_ and rSO_2_ and their uncoupled relationship in patients with diabetes indicate that disturbances in the cortical oxygen saturation cannot be predicted from the global clinical parameter, the ScvO_2_. Thus, our findings advocate the monitoring value of NIRS in T2DM.

## Introduction

Since the increasing prevalence of type 2 diabetes mellitus (T2DM) overwhelms the decreasing rate of diabetes-related cardiovascular complication^1,2^, there is an increasing burden for health care providers^1,3^. Currently 30–40% of the patients undergoing cardiovascular surgery have T2DM^4^, which can partly be attributed to a therapeutic inertia in intensifying treatment of T2DM to reach optimal glycemic, lipid and blood pressure control^5^. In long-term T2DM, the high perioperative morbidity and mortality^6,7^ can be partly attributed to the development of characteristic pathophysiological changes in the cardiovascular system. The structural and functional abnormalities in the vasculature^8^ compromise adaptation mechanisms, subsequently increasing the risk of postoperative organ dysfunction including perioperative cerebral circulatory deficiency^9,10^. The reduced vasodilatory reserve capacity increases the risk for a reduced cerebral tissue oxygen supply in T2DM patients, which may be responsible for the higher incidence of postoperative adverse neurocognitive dysfunction and stroke in the diabetic population^11–14^.

In the perioperative period, the oxygen balance is routinely estimated from the oxygen saturation of central venous blood (ScvO_2_)^15^. However, ScvO_2_ reflects the overall oxygen balance^16^. Since substantial heterogeneity exists in oxygen extraction of various organs^17^, the mixed venous oxygen saturation is not able to reflect regional changes in the tissue oxygenation. As the oxygen extraction rate of cerebral tissue is one of the highest in the body under physiological conditions^17^, ScvO_2_ bounds to overestimate cerebral tissue oxygen saturation^18^. Vascular dysfunction in T2DM may further impair the cerebral tissue oxygen balance^19^ and thus, the ability of ScvO_2_ to assess the intraoperative brain tissue oxygenation is particularly challenged in diabetic patients. Therefore, online monitoring of regional cerebral tissue oxygen saturation (rSO_2_) would have an advantage in T2DM patients in the intraoperative period to manage local hypoxemic episodes. Accordingly, we hypothesise that T2DM patients exhibit a significant widening of the gap between ScvO_2_ and rSO_2_ (gSO_2_), and ScvO_2_ is not suitable to infer rSO_2_ as a cardiovascular consequence of diabetes.

To test this hypothesis, we aimed at comparing direct measurements of rSO_2_ using near-infrared spectroscopy (NIRS) to simultaneously obtained ScvO_2_ data in patients with and without T2DM. ScvO_2_, rSO_2_ and gSO_2_ are expected to exhibit intraoperative changes during cardiac surgery depending on the patient management with or without cardiopulmonary bypass (CPB). Thus, measurements were made during anaesthesia in two groups of cardiac surgery patients: those undergoing CPB and those scheduled for off-pump coronary artery bypass (OPCAB) grafting procedure.

## Methods

### Patients

**Figure 1:**
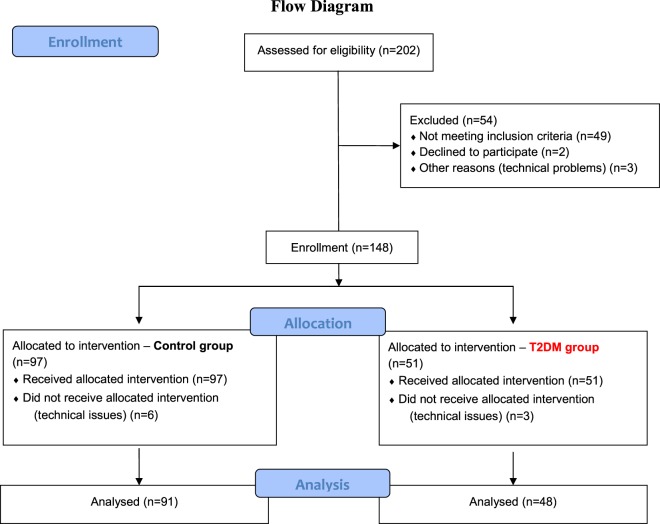
Patient flow chart.

Hundred thirty-nine consecutive patients with T2DM (n = 48) and control (C) subjects without T2DM (n = 91) undergoing cardiac surgery were enrolled in this prospective descriptive cohort study. Patients were defined as having T2DM if their medical history included a diagnosis of T2DM and haemoglobin A1c (HbA1c) > 6.4%. All patients underwent either OPCAB (C-OPCAB, n = 31 and T2DM-OPCAB, n = 24) or CPB (C-CPB, n = 60, T2DM-CPB, n = 24). Patients older than 80 years of age or with poor ejection fraction (<40%), unilateral internal carotid stenosis (>75%), or medical history of smoking, chronic obstructive pulmonary disease or stroke were excluded from the study. The protocol was approved by the Human Research Ethics Committee of Szeged University, Hungary (no. WHO 2788), and the patients gave their informed consent to participation in the study. The study was performed in accordance with the ethical standards laid down in the 1964 Declaration of Helsinki and its later amendments between January and August 2017. This manuscript adheres to the applicable CONSORT guidelines, and the patient flow chart is depicted on Fig. 1.

### Anaesthesia

One hour before the surgery, patients were premedicated with lorazepam (per os, 2.5 mg). Before induction of anaesthesia, the NIRS sensors (INVOS 3100, Somanetics, MI, USA) were positioned on both sides of the forehead. Sensors to detect depth of anaesthesia were also mounted on the forehead to monitor EMG and EEG activities. These signals were used to calculate response (RE) and state entropy (SE), respectively (GE Healthcare, Chicago, USA). Induction of anaesthesia was achieved by iv midazolam (30 μg/kg), sufentanil (0.4–0.5 μg/kg), and propofol (0.3–0.5 mg/kg), and iv propofol (50 mg/kg/min) was administered to maintain anaesthesia. Intravenous boluses of rocuronium (0.6 mg/kg for induction and 0.2 mg/kg every 30 minutes for maintenance) was administered iv to ensure neuromuscular blockade. A cuffed tracheal tube (internal diameter of 7, 8, or 9 mm) was used for tracheal intubation, and patients were mechanically ventilated (Dräger Zeus, Lübeck, Germany) in volume-controlled mode with decelerating flow. A tidal volume of 7 ml/kg and a positive end-expiratory pressure of 4 cmH_2_O were applied, and the ventilation frequency was adjusted to 12–14 breaths/min to maintain end-tidal CO_2_ partial pressure of 36-38 mmHg. Mechanical ventilation was performed with a fraction of inspired oxygen of 0.5 during the entire OPCAB procedure and before CPB, and it was increased to 0.8 after CPB. As a standard part of the cardiac anaesthesia procedure, oesophageal and rectal temperature probes were introduced, and a central venous line was inserted into the right jugular vein. The left radial artery was also cannulated to monitor systolic, diastolic and mean arterial (MAP) blood pressures and arterial blood gas samples.

The membrane oxygenator was primed with 1,500 ml lactated Ringer’s solution prior to CPB. Intravenous heparin (150 or 300 U/kg for OPCAB and CPB procedures, respectively) was injected into the patient, and an activated clotting time of 300 s was achieved during OPCAB and of 400 s during CPB procedures. During CPB, mild hypothermia was allowed, the mechanical ventilation was stopped, and the ventilator was disconnected without applying positive airway pressure. Before restoring ventilation, the lungs were inflated 3–5 times to a peak airway pressure of 30 cmH_2_O to facilitate lung recruitment. Normothermia was maintained in the OPCAB patients.

### Measurement of cerebral-tissue oxygen saturation

The spatially resolved continuous-wave NIRS technique was applied to estimate rSO_2_. This monitor uses two different wavelengths (730 and 810 nm) and has two detectors positioned 3 and 4 cm from the light source. Computing the differences between the intensity of the emitted and the reflected light^20^ with two receivers^21^ allows the measurement of the oxygen saturation of the cerebral cortex. In this study, two adult sensors were applied on the left and right sides of the patient’s forehead symmetrically, placed more than 3 cm from the superior rim of the orbit^22^.The cerebral-tissue oxygen saturation was monitored continuously during the surgical procedures and the data were registered in each protocol stage. The mean value of the rSO_2_ measured by the sensors was calculated for each protocol stage and used for further analyses.

### Measurement of central venous oxygen saturation and arterial blood gas parameters

The ScvO_2_ was measured from central venous blood samples (Radiometer ABL 505, Copenhagen, Denmark). The proper positioning of the central venous catheter was verified by the surgeon via manually palpating the catheter tip. The partial pressures of oxygen (PaO_2_) and carbon-dioxide (PaCO_2_), haemoglobin, pH and oxygen content (CaO_2_) were determined from arterial blood gas samples at each protocol stage.

### Measurement protocol

**Figure 2:**
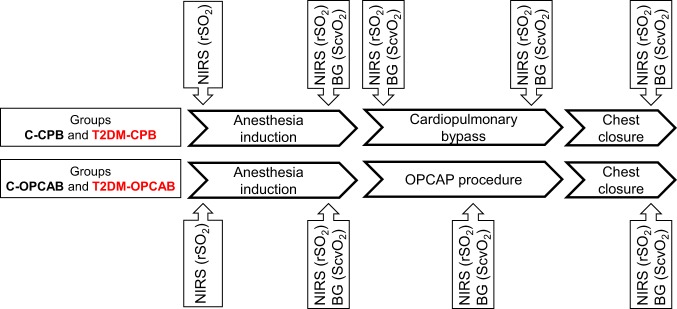
The scheme of the measurement protocol. Measurements were made at five time points in the patients with cardiac surgeries requiring cardiopulmonary bypass (Groups C-CPB and T2DM-CPB), while four measurements were performed in the patients with off-pump coronary bypass grafting surgeries (C-OPCAB and T2DM-OPCAB). Cerebral tissue oxygen saturation (rSO_2_) measured by near infrared spectroscopy (NIRS). Central venous oxygen saturation (ScvO_2_) determined from blood gas analyses (BG).

The scheme of the measurement protocol is outlined on Fig. 2. After securing arterial and peripheral venous lines and placement of NIRS and entropy sensors, data collection was initiated immediately before anaesthesia induction in all groups of patients. Since catheterization of the jugular vein was scheduled after anaesthesia induction, ScvO_2_ and gSO_2_ data were not available at the first protocol stage. After anaesthesia induction and muscle relaxation, all measurements were repeated before surgical incision. For the patients undergoing CPB procedures, the whole data set was registered at the beginning of CPB after clamping the aorta and 5 min before the end of CPB. For the patients undergoing OPCAB procedures, collection of the full set of data was performed during performance of the first proximal anastomosis between the aorta and saphenous vein graft. The final stage of the protocol was allocated to the end of the operation after sternal closure. All invasive (i.e. arterial and venous blood gas) and non-invasive data were registered simultaneously at each protocol stage after ensuring a 3 min steady-state condition.

### Statistical analyses

For numerically reported data, the scatters in measured variables are expressed as 95% confidence interval for the mean. The normality of the data was verified with the Shapiro-Wilk test; one-way analysis of variance (ANOVA) was used to test differences in the demographic, anthropometric, and clinical characteristics of the patients when they were included in the control, and diabetic groups. Fisher’s exact test was performed to assess the differences in the surgical procedures between the protocol groups. Pearson’s correlation test was applied to assess the relationship between global and regional oxygen saturation indices. Two-way repeated measures ANOVA with the inclusion of an interaction term was used for all measured variables with the protocol stage as within-subject factor (protocol stages) and group allocation as between-subject factor to establish the effects of T2DM and the surgical procedure on the oxygen saturation indices. The Holm–Sidak multiple comparison procedure was adopted to compare the variables in the study groups at different protocol stages. Further two-way repeated measures ANOVA tests were applied to assess between-group and within-group differences in the parameters influencing cerebral-tissue oxygen saturation. Sample sizes were estimated to enable the detection of a 10% difference in the primary outcome parameter gSO_2_ that we considered clinically significant. Accordingly, sample-size estimation based on an ANOVA test with four groups of patients indicated that 24 patients were required in each group to detect a significant difference between the protocol groups (the assumed variability of 10%, power of 80% and the significance level of 5%). Due to the prevalence of T2DM in cardiac surgery, this targeted number of T2DM patients resulted in twofold number of the patients without diabetes. The statistical tests were performed with the SigmaPlot statistical software package (Version 13, Systat Software, Inc., Chicago, IL, USA) and R environment. All reported p values are two-sided.

### Ethics approval and consent to participate

The protocol was approved by the Human Research Ethics Committee of Szeged University, Hungary (no. WHO 2788), and the patients gave their informed consent to participation in the study. The study was performed in accordance with the ethical standards laid down in the 1964 Declaration of Helsinki and its later amendments.

## Results

### Patient characteristics

Table 1 summarises the demographic, anthropometric and clinical characteristics and surgical procedures of the patients with and without T2DM. HbA1c was significantly higher in diabetic patients while there was no significant difference in the other parameters (i.e. weight, height, age or ejection fraction).

**Table 1:** Patient characteristics, surgical procedures and diagnoses (in parentheses).

		Group C (n = 91)	Group T2DM (n = 48)	p
Preoperative parameters	Weight (kg)	83 (79–86)	83 (77–89)	0.97
	Height (cm)	168 (166–170)	165 (163–168)	0.20
	Age (years)	65 (63–68)	68 (67–70)	0.30
	Ejection fraction (%)	60 (56–64)	56 (51–61)	0.15
	HbA1c (%)	5.6 (5.4–5.7)	7.2 (6.7–7.7)	<0.001
	Duration of T2DM (years)	—	11 (9–13)	—
Surgical procedures and diagnoses	ARR (AAA) (n)	1	0	1.00
	AVR (AS) (n)	28	9	0.15
	AVR (AI) (n)	1	1	1.00
	AVR + MVR (AS + MI) (n)	2	3	0.33
	AVR + CABG (AS) (n)	11	3	0.37
	AVR + CABG (AI) (n)	3	2	1.00
	MVR/P (MI) (n)	10	4	0.77
	MVR + CABG (MI + CAD) (n)	3	1	1.00
	OPCAB (CAD) (n)	31	24	0.07
	LA Myxoma (n)	1	1	1.00

HbA1c: Hemoglobin-A1c, ARR: aortic root replacement, AAA: aortic arch reconstruction; AVR: aortic valve replacement to repair aortic stenosis (AS) or aortic insufficiency (AI); MVR/P: mitral valve replacement/plasty; CABG: coronary artery bypass grafting; MI: mitral insufficiency; LA: left atrium, CAD: coronary artery disease. Data for preoperative parameters are shown as mean and 95% confidence interval for the mean; data for surgical procedures and diagnoses are represented as number of patients.

Serum glucose level, rSO_2_ and parameters determining tissue oxygen balance obtained before anaesthesia induction are demonstrated on Table 2. The serum glucose level was higher and the rSO_2_ values were lower in the T2DM patients as compared to the control patients. Evidence for a difference in parameters determining the oxygen supply was only observed in haemoglobin concentration and CaO_2_. Response entropy, state entropy and oesophageal temperature did not differ between the protocol groups.

**Table 2:** Intraoperative parameters before anaesthesia induction.

	Group C (n = 91)	Group T2DM (n = 48)	p
Glucose (mmol/L)	5.9 (5.7–6.0)	8.04 (7.2–8.9)	<0.001
rSO_2_ (%)	68 (66–69)	60 (58–63)	<0.001
MAP (mmHg)	88 (86–90)	86 (83–90)	0.29
CaO_2_ (ml/dl)	17.0 (16.4–17.5)	16.0 (15.2–16.7)	0.034
Hemoglobin (g/dl)	13.0 (12.7–13.5)	12.4 (11.9–13)	0.043
PaO_2_ (mmHg)	77.09 (71.70–82.48)	72.29 (69.11–75.46)	0.13
pHa	7.412 (7.408–7.417)	7.408 (7.396–7.420)	0.86
PaCO_2_ (mmHg)	38.27 (37.27–39.26)	37.88 (36.46–39.31)	0.66
RE	90 (83–96)	92 (86–97)	0.394
SE	86 (79–92)	91 (84–97)	0.33
Te (°C)	36.5 (36.2–36.7)	36.7 (36.2–37.0)	0.6
CPB duration (min)	83 (74–92)	89 (69–109)	0.625

The serum glucose level and initial cerebral tissue oxygen saturation (rSO_2_) with parameters determining tissue oxygen balance. MAP: mean arterial pressure; CaO_2_: arterial oxygen content; PaO_2_: arterial partial pressure of oxygen; PaCO_2_: arterial partial pressure of CO_2_; RE: response entropy; SE: state entropy, Te: esophageal temperature, CPB: cardiopulmonary bypass. Data are shown as mean and 95% confidence interval for the mean.

### Effects of T2DM on central venous and cerebral oxygen saturation during CPB and OPCAB procedures

**Figure 3:**
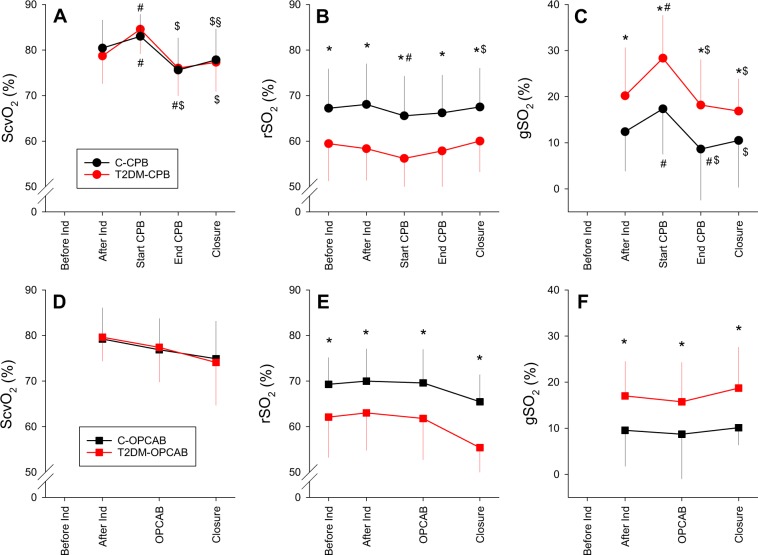
The central venous oxygen saturation (ScvO_2_, panels A,D), the cerebral oxygen saturation (rSO_2_, panels B,E) and the differences between these indices (gSO_2_, panels C,F) according to the protocol stages in patients with (red symbols) and without T2DM (black symbols) who underwent CPB (top panels) or OPCAB procedures (bottom panels). Error bars represent the following: SD. *p < 0.001 between the protocol groups within a stage, ^#^p < 0.05 vs. stage “After Ind.”, ^$^p < 0.05 vs. condition “Start CPB”, ^§^p < 0.05 vs. condition “End CPB” within a group.

Oxygen saturation in central venous blood (ScvO_2_), in cerebral tissue (rSO_2_) and their difference expressed as a saturation gap (gSO_2_), are presented in Fig. 3 for the patients underwent cardiac surgery with (top) or without (bottom) CPB. No significant difference was observed in ScvO_2_ between patients with and without T2DM at any protocol stage. Conversely, rSO_2_ was significantly lower in the T2DM-CPB and T2DM-OPCAB groups than in the corresponding controls (p < 0.001 for both), and this difference endured in all phases of the surgery. This result was reflected in significant differences in gSO_2_ between patients with and without T2DM in the CPB and OPCAB patients (p < 0.001 for both). During the surgical procedure, prominent and significant increases in ScvO_2_ (p < 0.001) with smaller but statistically significant decreases in rSO_2_ (p < 0.05) were observed at the beginning of CPB, resulting in marked elevations in gSO_2_ (p < 0.001). In both CPB groups, the ScvO_2_ and gSO_2_ reversed by the end of CPB, and these parameters decreased below their initial levels in C-CPB patients (p < 0.005). No significant intraoperative changes were observed in the oxygen saturation parameters in the OPCAB patients.

### Effects of T2DM on clinical parameters affecting cerebral oxygen supply and demand

**Figure 4:**
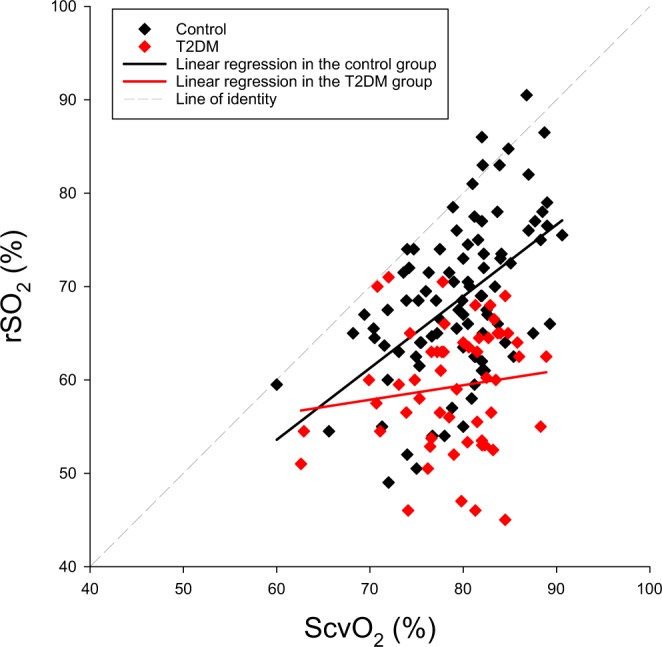
Correlation between cerebral oxygen saturation (rSO_2_) and central venous oxygen saturation (ScvO_2_) after the induction of anaesthesia in each patient. Linear regression in the T2DM group (p = 0.34, r = 0.13), Linear regression in the control group (p < 0.0001, r = 0.52).

Main clinical parameters characterizing tissue oxygen balance obtained at different stages of the protocol are summarised in Figs. 1S–4S in the Supplemental Digital Content. Between-group differences were observed only in the CaO_2_ before the surgical procedure (Fig. 1S, p < 0.05) in the blood glucose levels throughout the surgery (Fig. 4S, p < 0.05) and in the arterial oxygen saturation rSO_2_ differences (Fig. 2S, p < 0.05). Considerable within-group intraoperative changes were observed primarily during the CPB procedure. The onset of CPB was associated with small but significant decreases in core body temperature in both T2DM-CPB and C-CPB patients (Fig. 3S, p < 0.05), which subsequently returned to the initial value by the end of the surgery. Furthermore, MAP and CaO_2_ decreased significantly in both groups of patients when CPB was established (Fig. 1S, p < 0.05), and returned to their initial values following chest closure. RE and SE decreased significantly after anaesthesia induction in all groups of patients with no difference between patients with and without T2DM (Fig. 3S).

### Relationship between central venous and cerebral oxygen saturation: effects of T2DM

The relationship between central venous and cerebral oxygen saturation obtained after anaesthesia induction prior to the surgery for control and T2DM patients is illustrated in Fig. 4. Significant correlation was observed between ScvO_2_ and rSO_2_ in the control group (r = 0.52, p < 0.0001). In contrast, no significant correlation was found between these global and regional oxygen saturation parameters in patients with T2DM (r = 0.13, p = 0.34).

## Discussion

Significant reduction of brain tissue oxygen saturation was obtained in the present study in T2DM patients. Since this finding was not reflected in the central venous oxygen saturation, the gap between ScvO_2_ and rSO_2_ widened significantly and the relationship between these two values became uncoupled in patients with diabetes. In the T2DM patient, the oxygen saturation gap remained elevated throughout the cardiac surgery procedure.

The lower initial rSO_2_ (Fig. 3B,E) and the associated widening of gSO_2_ (Fig. 3C,F) in patients with T2DM cannot be attributed to differences in demographic, anthropometric and clinical characteristics between the two groups (Tables 1 and 2). In both groups of patients, the oxygen demand of the cerebral cortex was expected to be in a uniformly low range throughout the surgery, as suggested by the therapeutic entropy levels (Fig. 3S). The glucose levels differed between normal and diabetic patients (Table 2 and Fig. 4S), which may affect the light scattering in the near infrared wavelength range^23^. However, this effect was negligible at the applied wavelength^24^ or was markedly smaller (<1.5%) that the differences obtained between the study groups^23^. Therefore, this phenomenon may have had a minor contribution to our findings, but cannot fully explain the differences in rSO_2_ between the diabetic and control groups. The presence of anaemia and lower CaO_2_ in the T2DM patients (Table 2) may contribute to the differences in rSO_2_ between control and diabetic patients. However, ScvO_2_ did not differ between the protocol groups (Fig. 3), suggesting sufficient oxygen supply in all patients under general anaesthesia with muscle relaxation when the oxygen demand of the paralyzed skeletal muscle decreases markedly. Accordingly, the diminished overall oxygen extraction can be supplied both in patients with normal or compromised microcirculation (such as T2DM). Moreover, the widened gap in diabetic patients remains after CPB despite the lack of difference in CaO_2_ and MAP between diabetic and non-diabetic patients. Most probably, the compromised rSO_2_ can rather be explained by the adverse cerebrovascular consequences of T2DM. The pathologic metabolic milieu is characterised by hyperglycaemia, insulin resistance and elevated level of free fatty acids^25^. Blockade of the vasodilatory insulin signalling pathway diminishes endothelial nitric oxide (NO) synthesis^26^. Endothelial NO production is further compromised by the advanced glycation end-products^26^ and by the increased inactivation of NO by oxidative stress^26^. The reduced NO-mediated endothelium-dependent vasodilation increases the vascular tone. The elevated arterial tone and/or vascular remodelling facilitated by these mechanisms are associated with low-grade inflammatory, prothrombotic proliferative processes, resulting in atherosclerotic plaque formation^27^. All these mechanisms converge to an impaired microcirculation^28^, which may be reflected in impaired cerebral-tissue oxygen saturation.

The few previous results available for anaesthetised patients measured before surgical intervention revealed somewhat lower cerebral oxygen saturation in diabetic than non-diabetic patients^18,29,30^, or failed to demonstrate an effect of diabetes on this outcome^31^. The discrepancies between these earlier results and the current finding may be attributed to the relatively small number of patients involved in the previous studies. Considering the substantial inter-individual variability in our rSO_2_ data obtained in diabetic patients (Fig. 3), the statistical power may not have been robust enough to reach a conclusion with confidence in the previous studies. All these previous studies applied jugular venous bulb oximetry to assess cerebral oxygen saturation, which reflects the oxygen status of the entire brain, whereas NIRS utilised in the present study focuses on the cortex. Since both the blood flow and oxygen demand is higher in the cortex than in the white matter^17^, microvascular dysfunction and remodelling may be more apparent in the cortical region. The sole previous study where NIRS data were reported for a diabetic subpopulation was part of a preoperative assessment without evaluating changes in the intraoperative period^32^. In this study, diabetes was associated with lower baseline rSO_2_ in vascular surgery patients; however, patients undergoing cardiac surgery procedures exhibited no difference preoperatively. Nevertheless, the high ratio of smokers and inclusion of aged patients in this previous study may have blunted the distinct effects of diabetes on the measured outcomes.

Remarkable intraoperative changes were observed in ScvO_2_, rSO_2_ and gSO_2_ at the beginning of CPB (Fig. 3, top panel). The increases in ScvO_2_ can be attributed to a decreased core temperature (Fig. 3S) and subsequent decrease in the systemic oxygen demand^33^. The concomitant slight, but significant, decrease in rSO_2_ in the diabetic patients should be interpreted in terms of a decreased MAP and haemoglobin concentration associated with an elevation in arterial pH (Fig. 1S)^34^. The opposite changes in ScvO_2_ and rSO_2_ after the onset of CPB are reflected in the striking elevations in gSO_2_ (Fig. 3C). It is of note that in diabetic patients the gSO_2_ may rise to a value threefold greater than physiologically normal (i.e. from around 10 to around 30%, Fig. 3C). The compromised rSO_2_ observed in the present study may be responsible for the increases in postoperative adverse neurocognitive outcomes and stroke in T2DM patients^11–14^. In contrast with the CPB patients, no intraoperative changes in oxygen saturation indices were detected in the patients with OPCAB procedures (Fig. 3, bottom panel). This more stable pattern of cerebral-tissue oxygen saturation may explain the lower incidence of postoperative stroke^13,35^ and cognitive dysfunction^36^ after cardiac surgery with OPCAB.

The brain has high oxygen demand as well as low hypoxic tolerance and the regulation of cerebrovascular circulation is correspondingly complex^37^. Therefore, the oxygen saturation of this organ can distinctly differ from the rest of the systemic circulation. NIRS offers a simple, non-invasive, real-time monitoring tool to quantify the oxygen status of brain tissue. Hence, this technique has a great potential to reveal disturbances in the regional oxygen saturation promptly, with a particular advantage of assessing cerebral tissue oxygen saturation in the perioperative period. In non-diabetic patients, there is a significant difference between global and cerebral-tissue oxygen saturations, although they exhibit significant correlations (Fig. 4). Nevertheless, there is a scatter in this relationship due to recognized interindividual variability even in the control patients^38,39^. The scatter in the rSO_2_ data can be attributed to the relatively large age range (67.1 ± 12.2 for the whole population) and to the involvement of patients with wide variety of heart diseases to our cohort. Nevertheless, the associations between these regional and global oxygenation indices suggest the possibility to predict of the brain oxygen status from the ScvO_2_ value when the cerebral circulation is intact. Conversely, our results also demonstrate that in addition to the gap between ScvO_2_ and rSO_2_, these parameters became uncoupled in diabetic patients (Fig. 4). The lack of a clear relationship between these indices impedes the assessment of cerebral-oxygen saturation from ScvO_2_ in the T2DM population. Accordingly, our findings demonstrate the particular monitoring value of NIRS in the presence of a disease affecting cerebral microcirculation. These considerations may contribute to the clarification of the monitoring value of NIRS in a critically ill population, which is currently under debate^40–43^.

A methodological aspect of the study is related to the general limitations of the NIRS in the accurate assessment of cerebral oxygenation, such as lack of established baseline values, possible contamination by scalp tissue, localised reading and the assumption of fixed arterial-venous blood ratio of the tissue^40,44^. The INVOS is considered as a trend monitor. However, several studies based their conclusions on absolute values of the rSO_2_^45,46^. In our study, involvement of high number of patients enabled to detect statistically significant differences between the protocol groups with high statistical power, confirming the validity of our conclusions. Another methodological aspect concerns the interindividual variability in the cardiac surgery patients. However, the groups of the patients with and without T2DM did not differ in anthropometric parameters, diagnoses or surgical procedures, and the comorbidities with potential biasing effect on cerebral circulation were excluded. This study focused on the intraoperative changes of tissue oxygenation, further studies are required to follow-up potential differences in the clinical outcomes (e.g. stroke or neurocognitive dysfunction) between normal and T2DM patients.

## Conclusions

In conclusion, the present study demonstrated that diabetes mellitus worsens the oxygen saturation of the cerebral tissue and uncouples indices reflecting regional cortical and global central venous oxygenation. Consequently, disturbances in the cortical oxygen saturation in diabetic patients become unpredictable from the well-established global clinical parameter of central venous oxygen saturation. Thus, diabetic patients may benefit from the continuous intraoperative measurement of regional brain-tissue oxygen saturation to optimise tissue oxygenation with adjusting cerebral perfusion pressure, arterial oxygen content and/or avoiding alkalosis. Our findings may also contribute to the identification of patient populations with abnormal microcirculation when NIRS has particular monitoring value for detecting cerebrovascular adverse consequences.

## Supplementary information

Supplementary Information

## Data Availability

The datasets analysed during the current study are available from the corresponding author on a reasonable request.
