# Supplementary material for: Differences Between Central Venous and Cerebral Tissue Oxygen Saturation in Anaesthetised Patients With Diabetes Mellitus
Source: Sci Rep. 2019 Dec 24;9:19740. doi: 10.1038/s41598-019-56221-4 (PMC6930198; doi:10.1038/s41598-019-56221-4)
Supplement: Supplementary file 1 — Supplementary Information [file 41598_2019_56221_MOESM1_ESM.docx]

DIFFERENCES BETWEEN CENTRAL VENOUS AND CEREBRAL TISSUE OXYGEN SATURATION IN ANAESTHETISED PATIENTS WITH DIABETES MELLITUS

**Online data supplement**

^1, 2^Roberta Sudy, ^2^Ferenc Petak, ^1, 2^Almos Schranc, ^1^Szilvia Agocs,
^1^Ivett Blaskovics, ^3^Csaba Lengyel, ^1^Barna Babik

^1^Department of Anesthesiology and Intensive Therapy, University of Szeged, Szeged, Hungary

^2^Department of Medical Physics and Informatics, University of Szeged, Szeged, Hungary

^3^ 1st Department of Medicine, Medical Faculty, University of Szeged, Szeged, Hungary

**Figure 1S.** Intraoperative changes in the parameters determining cerebral-tissue oxygen supply in patients with (**n=48, red symbols**) and without (**n=91, black symbols**) diabetes mellitus. CPB procedures on the top, OPCAB procedures are on the bottom panels. MAP: mean arterial pressure: CaO_2_: arterial oxygen content; pHa: arterial pH. *: p<0.05 control vs. T2DM, +: p<0.05 vs. before induction, #: p<0.05 vs. after induction, $: p<0.05 vs. start CPB or OPCAB, §: p<0.05 vs. end CPB.

_
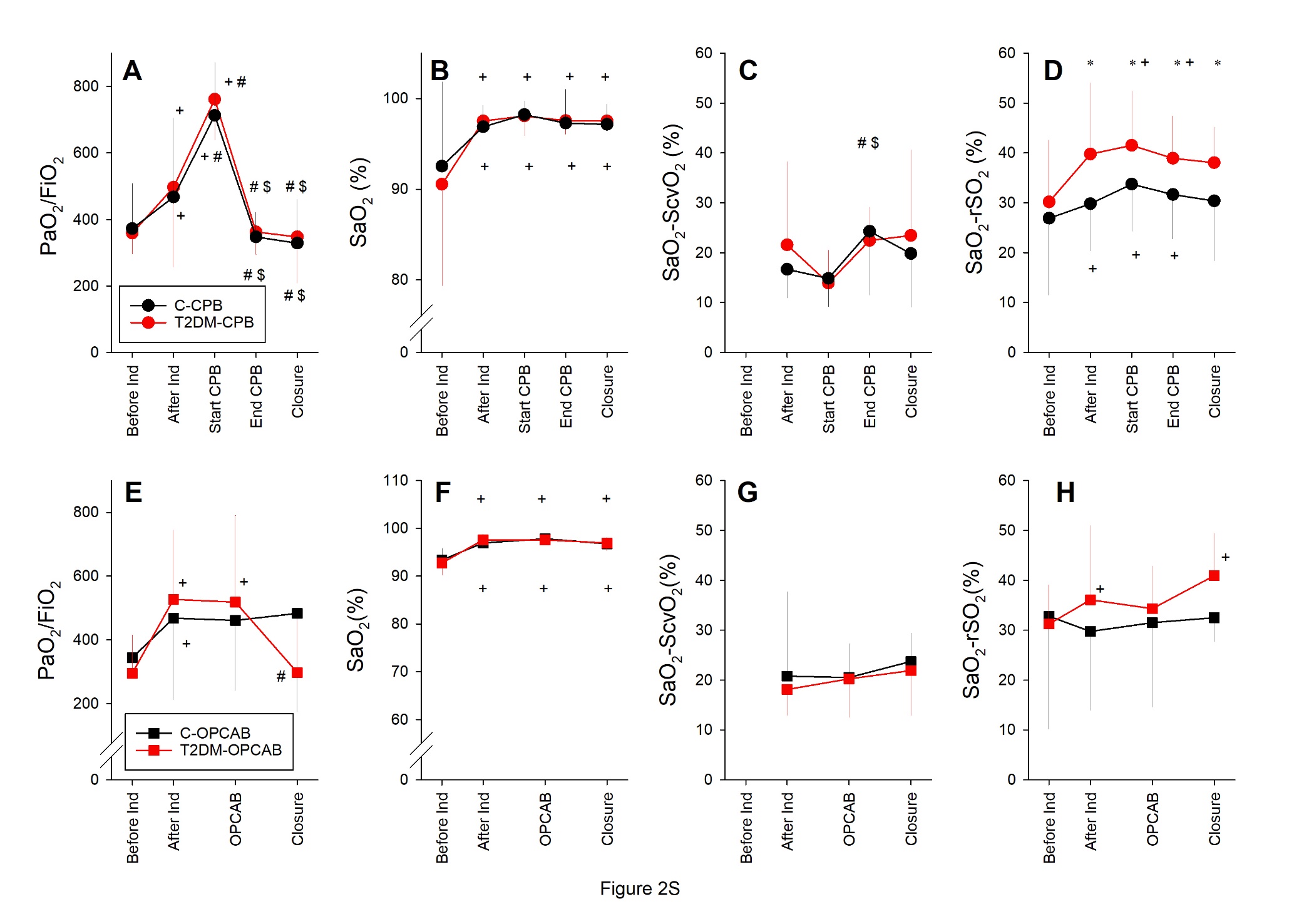
_**Figure 2S.** Intraoperative changes in the oxygenation index (PaO_2_/FiO_2_), arterial oxygen saturation (SaO_2_) and the differences between arterial and central venous (SaO_2_-ScvO_2_) and cerebral-regional staturation (SaO_2_-rSO_2_) (**n=48, red symbols**) and without (**n=91, black symbols**) diabetes mellitus. CPB procedures on the top, OPCAB procedures are on the bottom panels. *: p<0.05 control vs. T2DM, +: p<0.05 vs. before induction, #: p<0.05 vs. after induction, $: p<0.05 vs. start CPB or OPCAB.

**Figure 3S.** Intraoperative changes in the parameters determining cerebral-tissue oxygen demand in patients with (**n=48, red symbols**) and without (**n=91, black symbols**) diabetes mellitus. CPB procedures on the top, OPCAB procedures are on the bottom panels. Te: esophageal temperature: RE: Response Entropy; SE: State Entropy. +: p<0.05 vs. before induction, #: p<0.05 vs. after induction, $: p<0.05 vs. start CPB or OPCAB, §: p<0.05 vs. end CPB.

**Figure 4S.** Intraoperative changes in serum glucose levels in patients with (**n=48, red symbols**) and without (**n=91, black symbols**) diabetes mellitus. CPB procedures on the top, OPCAB procedures are on the bottom panels. *: p<0.05 control vs. T2DM, +: p<0.05 vs. before induction, #: p<0.05 vs. after induction, $: p<0.05 vs. start CPB or OPCAB.
